# Effectiveness and Safety Profile of Dupilumab in Chronic Rhinosinusitis with Nasal Polyps: Real-Life Data in Tertiary Care

**DOI:** 10.3390/ph16040630

**Published:** 2023-04-21

**Authors:** Cosimo Galletti, Maria Antonietta Barbieri, Francesco Ciodaro, Francesco Freni, Francesco Galletti, Edoardo Spina, Bruno Galletti

**Affiliations:** 1 Department of Adult and Developmental Human Pathology “Gaetano Barresi”, University of Messina, 98100 Messina, Italy; 2 Department of Clinical and Experimental Medicine, University of Messina, 98100 Messina, Italy

**Keywords:** dupilumab, safety profile, chronic rhinosinusitis with nasal polyps, real-word evidence, otorhinolaryngology

## Abstract

Chronic rhinosinusitis with nasal polyps (CRSwNP) is characterized by a type 2 pattern of inflammation resulting in the production of some cytokines. Dupilumab radically changes the treatment of CRSwNP, but, considering its recent approval, it may be useful to evaluate its safety profile in a real-world setting. This work aimed to prospectively highlight the effectiveness and safety profile of dupilumab in patients with CRSwNP enrolled in the Otorhinolaryngology Unit of the University Hospital of Messina. An observational cohort study was carried out considering all patients treated with dupilumab. A descriptive analysis was conducted reporting all demographic characteristics, endoscopic evaluations, and symptom conditions. A total of 66 patients were treated with dupilumab, but three patients were excluded due to a lack of adherence during the observational period. A statistically significant reduction in the Sino-Nasal Outcome Test 22 (SNOT-22) and nasal polyps score (NPS) was shown at the 6th and 12th months compared to baseline values (SNOT-22, −37 and −50, *p* < 0.001 for both comparisons; NPS, −3 and −4, *p* < 0.001 for both comparisons). During the follow-up, eight patients (12.7%) had a reaction at the site of injection, and seven (11.1%) had transient hypereosinophilia. Given the optimal treatment response and the minimal adverse effects observed, clinicians should consider dupilumab a safe and effective treatment. Further studies are necessary to better understand the long-term effects.

## 1. Introduction

Chronic rhinosinusitis (CRS) is a widespread disease, affecting approximately 5–28% of the population worldwide [1,2,3,4,5,6]. The last European Position Paper on Rhinosinusitis and Nasal Polyps (EPOS) explains that CRS consists of inflammation of the nasal mucosa and the paranasal sinuses, clinically characterized by two or more symptoms, one of which should be either nasal congestion or nasal discharge and/or facial pain, pressure and/or reduction/loss of smell and either endoscopic signs of nasal polyps and abnormalities such as discharge and swollen mucosa in the middle meatus and mucosal changes within the osteo-meatal complex and sinuses on CT scan of the sinuses lasting at least 3 months [6]. Overall, CRS is a clinic-based diagnosis verified by a classic nasal endoscopic exam and a head CT scan. CRS is classified as chronic rhinosinusitis with nasal polyps (CRSwNP) and without nasal polyps (CRSsNP). CRSwNP is evaluated in 1–4% of the general population and 25–30% of patients with CRS. The mean age of patients is between 40 and 60 years at the time of diagnosis, but the first symptoms often begin between the ages of 20 and 30. The prevalence of CRSwNP increases with increasing age and is half as prevalent in men as in women, with a sex ratio of 1.3 [6,7]. Most rhinological symptoms, such as nasal obstruction, anterior rhinorrhea, posterior rhinorrhea, sneezing attacks, heaviness or pain in the face, are encountered in all sinus diseases, acute or chronic. Anosmia and loss of taste are the only two symptoms with strong diagnostic value for sinus polyps. The study of the symptoms makes it possible to monitor the effectiveness of the proposed treatments. Since sinonasal polyps are a chronic disease of the airways, the patient will be followed up long-term, as with any chronic disease. Several methods of quantification have been proposed: the visual analog scale (VAS), DyNaChron questionnaire, severity class quantification and quality of life questionnaire. Furthermore, the anamnesis also looks for a history of allergy, asthma, chronic cough, intolerance to aspirin, nonsteroidal anti-inflammatory drugs (NSAIDs) or sulfites (wines) and otological manifestations (repetitive otitis, chronic otitis). Clinical examination should be performed with a flexible endoscope or a rigid endoscope. It is carried out in consultation with the seated patient, to whom the technique and inconveniences must be explained first. For most clinicians, it is not useful to practice local anesthesia or apply decongestants.

The diagnosis of sinonasal polyposis imposes two clinical criteria: the discovery of polyps in the nasal cavities with a yellowish-white “cluster of grapes” appearance, more or less inflammatory, within more or less abundant and more or less infected secretions and topographic criteria—since sinonasal polyps is a disease of the respiratory mucosa, its development is bilateral and more or less symmetrical. Polyps must be bilateral, more or less symmetrical. The presence of strictly unilateral polyps should cast doubt on the diagnosis of sinonasal polyps. These unilateral polyps testify usually to the existence of a local inflammatory process whose origin can be represented by an infection (fungal ball, for example) or by a tumor (inverted papilloma, for example). Sinonasal polyps is a chronic edematous disease of the sinonasal mucosa that affects both the anterior and posterior sinuses of the face. Thus, the presence of unilateral polyps originating from the middle meatus and sphenoethmoid recess signals damage to the entire unilateral ethmoid and should raise suspicion of a benign or malignant tumor. Several classifications of polyp volume have been proposed [8,9,10]. Each polyp has a highly variable size and has a peduncle with a more or less broad base. Polyps are marked by epithelial, vascular and matrix remodeling, as well as the presence of an inflammatory infiltrate in the stroma. The thickness of the epithelium is highly variable. The surface of the polyp involves a pseudostratified respiratory epithelium with hair cells and goblet cells. The percentage of hair cells decreases at the expense of goblet hyperplasia. There may be squamous metaplasia with more or less important keratinization. The epithelial cell proliferation index is higher in polyps than in normal nasal mucosa [11]. The stroma of the polyp contains a large variety of inflammatory cells: eosinophils, neutrophils, mast cells and lymphocytes. Two forms of sinonasal polyposis are conventionally distinguished: an eosinophil-rich sinonasal polyposis and an eosinophil-poor sinonasal polyposis. This classification corresponds to different immunological profiles based on nasal cytology [12]. The quality of life of these patients is very poor due to the sensorial loss and inflammatory affection of the upper and lower respiratory airways [2,3,6]. The association between asthma and CRS is strongly reported by the scientific literature: approximately 25% of patients with CRS compared to 5% of the general population. In particular, in CRSwNP patients, the association with asthma rises to 30–70%, and the NP condition is related to a more insidious pattern of asthma with higher severity [13,14]. 

CRS pathogenesis is based on both innate and adaptive immunity, but it also depends on mucus abnormalities and malfunctioning of the epithelial barrier [13,14,15]. Patients with CRS can be classified into three endotypes according to the presence of type 1, type 2 or type 3 inflammation, each regulating the expression of three different large cytokine clusters [16,17]. The deficient barrier function of the epithelium and the type 2 pattern of inflammation play a key role in the pathogenesis of CRSwNP, resulting in the production of some cytokines, including interleukin 4 (IL-4), IL-5 and IL-13. Both IL-4 and IL-13 activate the same heterodimeric receptor composed of two subunits, IL-4Rα and IL-13Rα1 chain [18]. Therefore, cytokines have become pharmacological targets for biological therapy. These new drugs increase the quality of life of patients with CRSwNP, control the underlying disease and minimize the side effects of chronic therapeutic protocols with oral corticosteroids. The first biologic approved for CRSwNP was dupilumab in 2019 [19]. Dupilumab is a fully human monoclonal antibody (mAb) approved in June 2019 by the US Food and Drug Administration (FDA) for the treatment of CRSwNP when systemic therapy with corticosteroids and/or surgery does not allow adequate control of the disease. Dupilumab binds specifically to the IL-4Rα receptor subunit and thus blocks both IL-4 and IL-13 signaling; consequently, dupilumab inhibits the cytokine/chemokine-induced response and IgE synthesis [18]. In addition, dupilumab transiently increases blood eosinophil concentrations by inhibiting eotaxin-3, resulting in a lack of migration of eosinophils from peripheral blood to polyp tissue [20]. Considering the safety profile established from premarketing studies, dupilumab seems to be well tolerated, with no serious adverse events (AEs) [21]. Recently, in 2020, omalizumab, an anti-IgE monoclonal antibody, was approved by the FDA also for CRSwNP thanks to evidence from the phase III clinical study Polips I and II. Omalizumab works by binding the Fc receptors of mast cells and basophils, reducing the total serum levels of IgE. At the beginning, in 2003, it was prescribed in patients with allergic asthma [19]. In the last few months, another therapeutic approach for CRSwNP has emerged, namely mepolizumab, an anti-IL-5 monoclonal antibody that regulates the eosinophil activity, decreasing the blood and tissue eosinophil counts, and it is approved for severe eosinophilic asthma (SEA), eosinophilic granulomatosis with polyangiitis (EGPA), hypereosinophilic syndrome (HES) and CRSwNP [19]. 

Considering that dupilumab was recently approved in Italy for the treatment of CRSwNP and is widely used in clinical practice, the awareness of its effectiveness and safety profile is not entirely clear. For all the above reasons, the aim of this study was to prospectively highlight the effectiveness and safety profile of dupilumab in patients with CRSwNP enrolled in the Otorhinolaryngology Unit of the University Hospital of Messina.

## 2. Results

A total of 66 patients were enrolled in the Otorhinolaryngology Unit of the University Hospital of Messina and considered for dupilumab therapy. During the observational period, three patients were excluded due to a lack of adherence to the treatment in accordance with the EPOS criteria. Of all 63 patients, 43 were men (68.3%) while 20 were women (31.7%), with a median (Q1–Q3) age of 54 (46–64) years. Fifteen patients (23.8%) were smokers (11 men and 4 women). Moreover, 42 patients (66.7%), namely 25 women and 11 men, had a history of allergic conditions. Concomitant asthma was present in 34 patients (54.0%), of which 19 were women and 15 were men. The median value of Sino-Nasal Outcome Test 22 (SNOT-22) pretreatment was 70 (58–78), with a median nasal polyps score (NPS) at pretreatment of 6 (5–7), which indicated severe CRSwNP symptomatology. At baseline, only two patients (3.2%) were not compliant with the therapeutic protocol and were excluded from the study. All patients were under treatment with intranasal corticosteroids and were unresponsive, and 46 (73.0%) were previously subjected to endoscopic sinus surgery (ESS). General characteristics of the sample size and post-surgical and naïve groups are reported in Table 1. In the post-surgical group, a higher percentage of males and the elderly was shown without any statistically significant differences compared to naïve patients, while a statistically significantly higher percentage of smokers was reported for naïve patients compared to post-surgical ones (43.5% vs. 10.9%, *p* = 0.005). No differences were observed for any concomitant conditions or endoscopic and symptom evaluations between the post-surgical and naïve groups.

**Figure 1:**
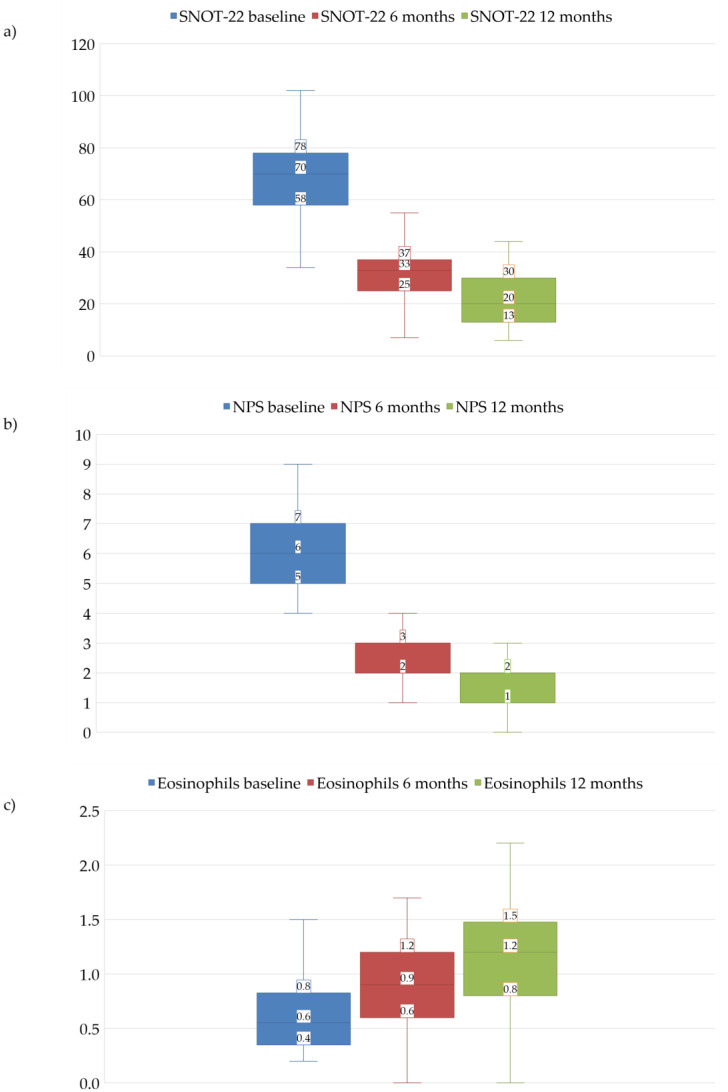
Median value variations over time: (**a**) SNOT-22, (**b**) NPS, (**c**) eosinophil count. SNOT-22 = Sino-Nasal Outcome Test 22; NPS = nasal polyps score.

All patients completed the 6- and 12-month follow-up. At the last follow-up, patients were in treatment with dupilumab with a median (Q1–Q3) of 12 (6–18) months. The median (Q1–Q3) SNOT-22 score at 6 months was 33 (25–37), while the median NPS score was 3 (2–3). The median (Q1–Q3) SNOT-22 score at 12 months was 20 (13–30), while the median NPS score was 2 (1–2) (Figure 1).

A statistically significant reduction in SNOT-22 and NPS was shown at the 6th and 12th months compared to baseline values (SNOT-22, −37 and −50, *p* < 0.001 for both comparisons; NPS, −3 and −4, *p* < 0.001 for both comparisons). The median baseline blood eosinophil count (×10⁹ cells per L) was 0.6 (0.4–0.8); during the follow-up visits, the blood eosinophil count was as follows: 0.9 (0.6–1.2) at the 6th month and 1.2 (0.8–1.5) at the 12th month (Figure 1). A statistically significant increase in blood eosinophil count was observed at the 6th and 12th months (+0.1 and +0.4, *p* < 0.001 for both comparisons) (Table 2).

At the 12-month follow-up, according to EUFOREA indications, all patients were considered to remain in treatment with dupilumab and continued the treatment because of a reduced NPS, improved quality of life and a reduced need for system corticosteroids (good response 3–4 criteria).

### Safety Profile

Dupilumab seemed to be well tolerated by all patients. However, during the follow-up at the third month, eight patients (12.7%) reported that they had an ADR at the site of injection referring to rubor, calor and dolor during the first 3 days after the injection. The patients were treated with betamethasone dipropionate and gentamicin sulfate, with a local application two times a day until the end of symptomatology. Moreover, two patients (3.2%) had monoarticular arthralgia shortly after the second injection, while one patient (1.6%) reported pyrexia on the third day after the second injection. These two AEs were controlled with NSAIDs and/or paracetamol 1 gr oral cpr as needed until complete resolution. Another patient (1.6%) had monolateral conjunctivitis 5 months after the beginning of the treatment with dupilumab. The conjunctivitis was treated with topical corticosteroids and antibiotic drops after ophthalmologist consultation, with complete resolution after 3 days of treatment. Seven patients (11.1%) had transient hypereosinophilia confirmed by an increase in blood eosinophil count with stabilization and/or resolution: in detail, five patients had an increase at the 3-month follow-up visit, one at the 6-month follow-up visit and one at the 12-month follow-up visit. No changes in therapeutic protocol were needed; in fact, no AEs led to the cessation of biological therapy with dupilumab.

## 3. Discussion

The CRS pathogenesis is based on both innate and adaptive immunity, but it also depends on mucus abnormalities and malfunctioning of the epithelial barrier [13,15]. Patients with CRS can be classified into three endotypes according to the presence of type 1, type 2 or type 3 inflammation, each regulating the expression of three different large cytokine clusters [16,17]. The deficient barrier function of the epithelium and the type 2 pattern of inflammation play a key role in the pathogenesis of CRSwNP, resulting in the production of some cytokines, including IL-4, IL-5 and IL-13. Both IL-4 and IL-13 activate the same heterodimeric receptor composed of IL-4Rα and IL-13Rα1, which are expressed in a wide range of cells, including hematopoietic and non-hematopoietic cells, and they take part in several pathways: impairing the differentiation of keratinocytes, inducing the activation of eosinophils, increasing the production of fibroblasts by eotaxin and B cells by IgE and Th2 cell differentiation and survival [22,23]. Therefore, cytokines have become a pharmacological target for biological therapy. Dupilumab is a fully human monoclonal antibody (mAb) approved in 2017 by the US Food and Drug Administration (FDA) for the treatment of moderate-to-severe atopic dermatitis and moderate-to-severe asthma; in June 2019, dupilumab was the first mAb to gain FDA approval for the treatment of CRSwNP and was subsequently approved by the European Medicines Agency (EMA) for the treatment of CRSwNP when systemic therapy with corticosteroids and/or surgery does not allow adequate control of the disease [24]. Subcutaneous dupilumab is an add-on treatment to topic corticosteroids that is well tolerated. Dupilumab binds specifically to the IL-4Rα receptor subunit and thus blocks both IL-4 and IL-13 signaling; consequently, dupilumab inhibits the cytokine/chemokine-induced response and IgE synthesis [18]. The authorization was obtained following the results of the phase III studies LIBERTY NP SINUS-24 and LIBERTY NP SINUS-52, which demonstrated the efficacy and the safety of dupilumab 300 mg subcutaneously at weeks 24 and 52, respectively. Dupilumab reduces IgE, periostin, eotaxin-3 and thymus and activation-regulated chemokine concentrations [14]. Worsening of nasal polyps, a need for nasal polyp surgery or the use of systemic corticosteroids or both, headache, progression of asthma and epistaxis were more common with a placebo [14]. The diagnosis of CRSwNP leads to worsening in patients’ quality of life due to the sensorial loss and inflammatory effects on the upper and lower respiratory airways [2,3,6]. Since CRSwNP is a chronic disease of the airways, the patient will be followed up long-term, as with any chronic disease with a burden of disease that has significant healthcare-related costs [25]. For years, the treatment of CRSwNP was based on oral corticosteroids, often with antibiotics. However, patients showed persistence or recurrence of symptomatology [26]. Consequently, the study of this complex symptomatology makes it possible to monitor the effectiveness of the proposed treatments. Dupilumab has radically changed the treatment of CRSwNP when systemic therapy with corticosteroids and/or surgery does not allow adequate control of the disease [24].

Dupilumab started to be prescribed to patients affected by CRSwNP who were not responsive to the classic medical–surgical approach in January 2021 in the Otorhinolaryngology Unit of the University Hospital of Messina. During the COVID-19 pandemic, nasal surgeries for all non-malignant diseases, including CRSwNP, were strictly deprioritized. Consequently, some naïve patients with uncontrolled severe CRSwNP started dupilumab. Approximately 73% of patients were treated with at least one previous sinus surgery, and the other group was not fit for surgery due to medical–clinical conditions or due to the patient’s decision not to undergo functional endoscopic sinus surgery. No differences in terms of general characteristics were shown at baseline. Thus, we confirmed a comparable improvement in patients affected by severe CRSwNP during dupilumab treatment regardless of whether they had received surgery before. Moreover, sinus surgery is not always able to achieve long-lasting outcomes, with the recurrence of polyps ranging from 38 to 60% [2,27].

To determine the best medical or surgical treatment, recurring evaluation of the patient’s complaints is imperative to judge the effectiveness of the treatment. Descriptive results showed that dupilumab is effective in severe rebellious CRSwNP: a statistically significant reduction in SNOT-22 and NPS was shown at the 6th and 12th months compared to baseline values, according to the results found in the literature [2,3,5,6,14,28,29]. This could again support the gradual improvement in the quality of life of dupilumab-treated patients with increasing SNOT-22 changes and the improvement of sinonasal symptoms with increasing NPS changes. However, it should be noted that the results in terms of improvements in NPS and SNOT-22 were better in our study population, decreasing by more than 4 and 50 points, respectively. This could be attributed to the fact that most of the enrolled patients underwent functional endoscopic surgery of the paranasal sinuses, reestablishing ventilation of these structures and removing the ostial obstruction but also offloading the extrinsic inflammatory load in the affected sinuses [30]. All patients continued the treatment with dupilumab after 1 year of observation because of the reduced NPS, improved quality of life and reduced need for system corticosteroids; the evaluation of the treatment corresponded to three criteria and it was considered as a good response in accordance with the EUFOREA response criteria [31]. Furthermore, a statistically significant increase in blood eosinophil count was shown in the 6th and 12th months, according to the results reported by De Corso et al. [3] and Kariyawasam [18]. This condition is attributable to the mechanism of action of dupilumab, which transiently increases blood eosinophil concentrations by inhibiting eotaxin-3, resulting in a lack of migration of eosinophils from peripheral blood to polyp tissue [32]. In this sense, a stronger collaboration with rheumatologists and pulmonologists should be evaluated to implement decisions regarding therapy continuation [33]. However, most patients had no clinical symptoms, and most cases were mild to moderate, not requiring therapy discontinuation [34].

Considering the safety profile established from premarketing studies, dupilumab seems to be well tolerated, with no serious adverse events (sAEs) [29]. In CRSwNP studies, the most frequently reported AEs were nasopharyngitis, injection-site erythema, conjunctivitis, keratitis, cough, bronchitis and arthralgia [2,3,6,13,14,35]. Safety analysis confirmed that dupilumab was well tolerated, with a poor manifestation of AEs that completely resolved after symptomatological treatments. However, the rate of injection-site reactions was higher than that observed in the LIBERTY trials (12.7% vs. 6%) [14]. This could be attributable to more administration-related errors during auto-injection at home. Moreover, a significant association was shown between dupilumab injection and cases of arthralgia and joint swelling, generally occurring after the first month of treatment, as observed in one patient experiencing arthralgia at the second injection. The underlying mechanism is poorly understood, but it could involve the enhancement of IL-23 and IL-17 cell-mediated entheseal inflammation [36]. In addition, conjunctivitis is a well-known ophthalmic AE in dupilumab-treated patients that requires a detailed eye examination [33,37]. Another study found that the incidence of the AEs above was similar in patients with and without comorbid asthma; however, acute sinusitis and epistaxis were mostly reported when asthma was not a comorbidity in CRSwNP [21]. Furthermore, the first case of a cutaneous rash as a side effect of dupilumab in a patient with CRSwNP and asthma was shown; it may be related to the prevalent phenotype of type 2 inflammation in CRSwNP but also in asthma, which may induce an immunological imbalance resulting in dermatological side effects [38,39].

### Strengths and Limitations

The study has some strengths and limitations. Among the former, there is the prospective nature of data collection in a real-world setting, as already known in previous studies overcoming the efficacy–effectiveness gap with clinical trials [40,41,42]. Monoclonal therapy with dupilumab is effective in the control of type 2 inflammation leading to CRSwNP, supporting the indications of the AIFA for biological treatment with dupilumab. The sample size reported pieces of evidence from a real-life context; the established indication criteria, treatment protocol and follow-up standards for all patients strengthen the methodology. Moreover, a formation program for the patients during the first month of treatment was carried out to explain to them how to auto-inject the drug at home. The adherence rate was very high. Indeed, it was possible to focus on the effectiveness and safety because no changes were made in the administration plan over the first year of treatment. However, patients were followed by tertiary care that included the most severe and difficult-to-treat CRSwNP with an important history of rebellious and uncontrolled nasal polyposis. Other outcomes could not be evaluated, including olfactory function with Lund–Mackay scores; however, the SNOT-22 and NPS are valid tools to evaluate CRSwNP patients’ quality of life. Multicenter studies with a larger number of real-life patients enrolled and a longer observation time are necessary to confirm the effectiveness and safety profiles in these patients, especially in the long term. Indeed, an efficacy–effectiveness gap could become more evident due to predictive factors such as comorbidities, co-prescriptions of other drugs and the severity of the disease.

## 4. Materials and Methods 

### 4.1. Study Population

A monocentric observational cohort study was carried out to assess the effectiveness and safety of dupilumab in patients affected by CRSwNP who were followed by the Otorhinolaryngology Unit of the University Hospital of Messina, Italy, from January 2021 to January 2023. The sample size included all patients ≥18 years with a diagnosis of CRS and a minimum NPS of 4 who had received systemic and/or topical corticosteroids in the preceding two years, with previous sinonasal surgery or not. The exclusion criteria were low adherence to drug use, radio-chemotherapy treatment in the last 12 months, concomitant long-term systemic corticosteroid therapy for chronic autoimmune disease and pregnancy. A patient-encrypted code was used to maintain the anonymity of patients, in agreement with the Declaration of Helsinki.

### 4.2. Clinical Evaluation

The recorded characteristics were age (at first visit for dupilumab application), sex, smoking habits, history of allergic conditions, concomitant asthma, prior surgery, previous corticosteroid treatment, start date of dupilumab therapy and number of doses of dupilumab up until the dates of AEs. Patients were evaluated before starting the biological therapy and every six months with a general anamnesis, utilizing the SNOT-22 questionnaire and performing an endoscopic sinonasal evaluation to determine the NPS. A blood test with complete blood counts to evaluate the total serum immunoglobulin E (IgE) and eosinophil count was performed before starting the treatment and every six months. The date of the first dupilumab prescription during the study period was considered the “index date” for each patient. The prescription of dupilumab was in accordance with the criteria validated by the Italian Medicines Agency (AIFA) for CRSwNP treatment. The adherence to therapy was evaluated in accordance with the EPOS 2020 criteria, in which the panel advises to use dupilumab in patients with CRSwNP fulfilling the EUFOREA consensus for treatment with monoclonal antibodies [6,31]. Patients were subjected every 14 days to an injection of dupilumab 300 mg and underwent scheduled follow-up visits with the evaluation of clinical scores to establish the state of activity of CRSwNP, evaluating the reduction in NPS by endoscopic exam and considering the subjective perception of the disease using SNOT-22. The endoscopic exam was performed by evaluating each nasal fossa separately in accordance with NPS from 0 to 4 (0 = no polyps; 1 = small polyps in the middle meatus not reaching below the inferior border of the middle turbinate, 2 = polyps reaching below the lower border of the middle turbinate, 3 = large polyps reaching the lower border of the inferior turbinate or polyps medial to the middle turbinate and 4 = large polyps causing complete obstruction of the inferior nasal cavity). The total for both nasal cavities was registered as the NPS. The subjective perception of the disease was calculated using the Italian version of the SNOT-22, with a possible total score range from 0 to 110. Moreover, all AEs were collected during the follow-up period every three months. Each patient informed their clinician of any new symptoms that they may be experiencing since the start of dupilumab. The minimum follow-up period was six months. 

### 4.3. Data Analysis 

A descriptive analysis was performed using StatPlus:mac. Medians with interquartile ranges (Q1–Q3) were estimated for continuous variables, while absolute and percentage frequencies were estimated for categorical variables. Normality of variables was verified with the Kolmogorov–Smirnov test for normality. Since a non-normal distribution of some of the numerical variables was verified, a nonparametric approach was adopted. Groups of post-surgical and naïve patients were compared for baseline characteristics. All endoscopic evaluations by NPS and subjective perception comparisons by SNOT-22 were made between data obtained at different follow-up times (e.g., 6 and 12 months after the beginning of therapy) and baseline. The Wilcoxon test for dependent samples was performed to compare continuous variables, while Fisher’s exact test was used for qualitative variables. Statistical significance was assumed for *p* values < 0.05.

## 5. Conclusions

Dupilumab has recently been approved in Italy for the treatment of CRSwNP. This study seems to confirm the effectiveness and safety profiles of dupilumab in a real-world setting. Given the good treatment response and the minimal AEs observed, clinicians should consider dupilumab in CRSwNP regardless of previous surgery. Further studies are necessary to better understand the long-term effects of such monoclonal therapy in controlling CRSwNP.

## Figures and Tables

**Table 1:** Patients’ main clinical characteristics and endoscopic and symptom evaluations at baseline for the whole group and for the post-surgical and naïve groups separately.

	Post-Surgical (*n* = 46)	Naïve (*n* = 23)	*p* Value	All (*n* = 63)
Sex, male *n* (%)	34 (73.9)	9 (39.1)	0.112	43 (68.3)
Age, years median (Q1–Q3)	63 (49–69)	53.5 (45.3–58.3)	0.074	54 (46–64)
Smokers, *n* (%)	5 (10.9)	10 (43.5)	0.005	15 (23.8)
Allergic conditions, *n* (%)	27 (58.7)	15 (65.2)	0.794	42 (66.7)
Concomitant asthma, *n* (%)	22 (47.8)	12 (52.2)	0.932	34 (54.0)
SNOT-22, median (Q1–Q3)	74.5 (57.8–78.3)	63 (56.5–74.5)	0.224	70 (58–78)
NPS, median (Q1–Q3)	6 (5–7)	5 (5–6)	0.361	6 (5–7)
Blood eosinophil count, median (Q1–Q3)	0.5 (0.4–0.8)	0.8 (0.5–0.9)	0.077	0.6 (0.4–0.8)
Previous corticosteroid treatment, *n* (%)	46 (100)	23 (100)	-	63 (100)
Duration of therapy, median (Q1–Q3)	12 (12–18)	12 (12–15)	0.811	12 (6–18)

SNOT-22 = Sino-Nasal Outcome Test 22; NPS = nasal polyps score.

**Table 2:** Differences from baseline to each follow-up for SNOT-22, NPS and blood eosinophil count.

	6th Month vs. Baseline	*p* Value	12th Month vs. Baseline	*p* Value
SNOT-22, median (Q1–Q3)	−37	<0.001	−50	<0.001
NPS, median (Q1–Q3)	−3	<0.001	−4	<0.001
Blood eosinophil count, median (Q1–Q3)	+0.1	<0.001	+0.4	<0.001

SNOT-22 = Sino-Nasal Outcome Test 22; NPS = nasal polyps score.

## Data Availability

Data available within article.
